# Supplementary material for: Are carbon emissions trading and green financial instruments synergistic? -Comprehensive quantitative research based on content analysis
Source: PLoS One. 2024 Mar 7;19(3):e0298601. doi: 10.1371/journal.pone.0298601 (PMC10919601; doi:10.1371/journal.pone.0298601)
Supplement: S1 Data — (ZIP) [file pone.0298601.s001.zip › Data/地级市Z_F工作报告.zip!/╣π╢1⁄2/├╖╓▌╩╨/└┤╫╘╣1⁄2╓┌║┼í╛┬φ┐╦╩2╛▌═°í┐_2019.docx]

各位代表：

现在，我代表市人民政府向大会作政府工作报告，请予审议，并请政协各位委员和其他列席人员提出意见。

一、2018年工作回顾

2018年是我国、我省发展进程中不平凡的一年，也是梅州提振精气神、迈向新征程的重要一年。一年来，在市委的坚强领导下，在市人大常委会和市政协的监督支持下，市政府坚持以习近平新时代中国特色社会主义思想和党的十九大精神为指导，全面贯彻习近平总书记对广东重要讲话和重要指示批示精神，按照省委、省政府和市委的决策部署，综合施策、相机调控，保持经济社会健康发展，推动全市朝着全面建成小康社会目标迈出了新的步伐。全市地区生产总值完成1110.21亿元，增长2.4%；全体居民人均可支配收入21217元，增长8.1%；城镇新增就业2.58万人，年末城镇登记失业率2.27%；居民消费价格涨幅控制在1.7%。

（一）全力抓发展，努力稳定经济运行势头。我们积极采取措施，应对经济下行压力。持续扩大有效投资，梅汕高铁进入全线铺轨阶段，龙梅高铁、瑞梅铁路前期工作有序推进。5条在建高速公路加快推进，完成国省道新改建及路面改造163公里。葵岗立交互通工程顺利开工，客都大桥顺利通车。梅畲快线主体工程完工。梅县机场旅客吞吐量突破50万人次，增长29%。韩江高陂水利枢纽工程完成一期工程，梅州（五华）抽水蓄能电站主体工程全面动工建设。信息基础设施项目建设扎实推进，光纤接入用户突破百万，入户率达85%。社会消费品零售总额726.49亿元，增长8.7%。电商交易额、快递业务收入，分别增长17.1%和20.2%。“广东梅州金柚”项目被授予“2018年全国快递服务现代农业金牌项目”，业务量超过千万件。发展全域旅游，旅游接待总人数增长11.1%，旅游总收入增长13.3%。外贸稳步发展，实现进出口总额135.2亿元、增长1.9%。加快经济结构调整，三次产业比重调整为17.7∶32.1∶50.2。梅兴华丰产业集聚带加快建设，广梅园等园区加快提质增效，全市规上工业增加值193.12亿元，新增规上工业企业69家，规上工业利润同比增长37.8%，高技术制造业、先进制造业增加值占规上工业比重分别提高4.0、3.3个百分点。全社会用电量和工业用电量，分别增长10.7%和11.0%。民营经济加快发展，增加值增长3.5%。全年共引进亿元以上项目79个，承接珠三角地区产业梯度转移项目46个。新开展技术改造企业110家，新认定高新技术企业82家。金融支持实体经济力度加大，全年新增贷款187.16亿元，社会融资规模增量达249.64亿元，存贷比为54.94%、提高6.07个百分点，新增1家新三板挂牌企业。第四次全国经济普查前期工作扎实开展，维护统计数据真实性的要求得到较好落实。

（二）打好三大攻坚战，三年行动开局良好。我们坚决打好防范化解重大风险攻坚战，加强政府债务管理，全年无新增隐性债务、无风险预警。金融生态环境总体优良，在全省率先完成农信社改制农商行任务，率先实现村镇银行县域全覆盖，客家村镇银行成为全国首批“多县一行”制村镇银行试点。坚决打好脱贫攻坚战，突出产业和就业扶贫，选派5400多名驻村帮扶干部，累计筹集扶贫资金36.9亿元，实施产业扶贫项目10.9万个，转移就业1.82万人，全市5.28万户14.2万人实现脱贫，脱贫率94.12%。坚决打好污染防治攻坚战，中央环保督察交办案件全部办结，省环保督察交办案件办结率91.1%。全年梅州城区环境空气质量优良率98.9%，居全省第一。河（湖）长制有效落实，“五清”行动扎实开展。全面完成市级集中式饮用水水源地违法问题整治，县级及以上饮用水水源地水质达标率100%。5个城市黑臭水体整治成效明显，市域内国考和省考断面水质优良率100%。建成镇村生活污水处理设施137座、新开工228座，完成镇级填埋场年度整治任务。率先在粤北地区建成运营环保能源发电项目。农业面源污染治理得到加强。国家园林城市、国家卫生城市通过复审，全国文明城市、国家森林城市创建工作扎实推进。完成森林碳汇工程20.25万亩、生态景观林带工程168.5公里建设。

（三）全面深化改革开放，创新活力有效激发。我们盯住重点领域、关键环节，扎实推进51项重点改革事项。编制行政权力目录清单，清理规范市县行政职权，市政府部门权责清单事项压减162项，取消涉企涉民证明32项。清理规范行政审批中介服务9项，建成运营网上中介服务超市，进驻中介服务机构55家。落实鼓励高新技术、改善民生、支持小微企业发展政策减免税共14.38亿元。深化商事制度改革，实行“多证合一”改革，新增各类市场主体4.5万多户，增长35%。深化基本建设项目并联审批，企业开办时间压减至5个工作日内。深化政务服务“马上办”改革，加快推进“数字政府”改革建设，“粤省事•梅州”移动服务平台建设速度全省最快、上线特色事项全省最多。深化医药卫生体制改革，公立医院全面取消药品加成和医用耗材加成，规范公立医院薪酬制度，104个基层医院与市县医院共建医联体。乡村振兴综合改革成为全省试点。基本完成农村土地确权登记颁证任务。扩大对外开放，积极融入粤港澳大湾区建设，新设海外经贸文化联络处1家、总量达16家。综合保税区申报进入国家审批阶段，市跨境电商清关中心试运营。发挥创新支撑引领作用，国家级高新区、国家级经开区创建工作扎实推进，新增省级企业技术中心4家、省级工程中心22家、省重点实验室2家、院士工作站4家，建成1个国家级、1个省级小型微型企业创业创新示范基地，梅州互联网创新中心挂牌成立，30%以上的规上工业企业建立研发机构。

（四）加大统筹力度，城乡协调发展步伐加快。我们坚持以城带乡，加快城乡一体化发展。稳步推进嘉应新区起步区建设，基本建成5个安置区，建成江南新城18条主要道路管廊和芹洋半岛市政路网，马鞍山公园、芹洋湿地公园、剑英公园改造提升等项目建成使用，68个产业项目加快推进，学校、医院等公共配套设施不断完善。新城水厂首期日供水10万吨项目建成运行，城区用水难问题有效缓解。实施城市更新“微改造”，完成城区3条历史文化街区升级改造。各县县城扩容提质、中心镇“六个一”和特色小（城）镇建设步伐加快，新增4个省级特色小镇创建对象。开展“美丽梅州•美好家园”城乡环境大提升行动，实施整治违法建设“雷霆行动”，获评全国“2018美丽山水城市”。实施乡村振兴战略，农林牧渔业实现增加值198.96亿元、增长4.9%，5个现代农业产业园入选省级名单，新增省重点农业龙头企业17家、家庭农场214家，组建金柚等5个产业科技服务专家团队，省级农业龙头企业和省名特优新农产品数量均居全省第一。粮食、蔬菜、肉类等生产供应稳定。坚持兴林富民，新增省级以上林下经济示范基地8个。实施“客家菜师傅”工程，建成一批培训基地和食材供应基地，梅县玉水村获全省首个“广东厨师之乡”称号。全域推进农村人居环境整治，73%的自然村基本完成“三清三拆三整治”任务，梅县桃尧的经验做法得到央视《焦点访谈》栏目推广。农村生活垃圾有效处理率达96.8%，村庄保洁覆盖面达100%，其中349个省定贫困村达到干净整洁村标准。完成116公里山区中小河流治理、355宗村村通自来水工程建设和1453处削坡建房搬迁治理，完成农村危房改造任务。加快推进“四好农村路”建设，加宽改造窄路基路面687公里，建设公路安全生命防护工程2873公里。垦造水田项目全面开工，拆旧复垦验收面积居全省第一。

（五）千方百计改善民生，社会事业全面进步。我们坚持以人民为中心，民生投入占财政支出的八成以上，十件民生实事基本完成。城乡低保等6项底线民生保障水平不断提高，基本养老保险、医疗保险覆盖率分别达97.1%、100%，失业人员再就业2.46万人。民政救助、慈善、志愿服务、残疾人等工作成效明显。新开工城市棚户区1380套（户）、基本建成5535套（户）、发放租赁补贴621户。全面完成教育强镇复评，7个县（市、区）通过“广东省推进教育现代化先进县（市、区）”验收；动工建设8所公办幼儿园，建成4所公办中小学校；被省列为“职业教育综合改革示范市”创建单位；嘉应学院入选粤东西北高校振兴计划，得到中山大学结对帮扶。客家文化（梅州）生态保护实验区建设扎实推进。基层综合性文化服务中心基本实现行政村全覆盖。打造了山歌剧《春闹》等一批文艺精品。加强南粤古驿道保护利用，234公里古驿道纳入省重点线路，成功开发平远梅州岃等精品路段。加快创建卫生强市，推进28家县级医院、中心卫生院升级改造，完成1888间村卫生站建设，新增2家三甲医院，市人民医院入选广东省高水平医院建设单位。启动创建国家中医药综合改革试验区，县级二甲中医医院、基层医疗卫生机构中医馆均实现全覆盖；五华县、平远县被命名为县级全国基层中医药工作先进单位。群众体育蓬勃发展，完成1个、动工9个公共体育场馆建设，建成14个社区体育公园，建设城乡15分钟健身圈。我市青少年参加国家级竞技体育赛事获冠军4个、亚军6个。参加省运会团体总分取得历史性突破，梅州女足首次夺冠。加快足球改革发展，培育形成“两甲一超”职业球队格局，成为全国青少年校园足球改革试验区。高水平推进平安梅州、法治梅州建设，获评“2018全国社会治理创新示范市”，群众安全感和公安工作满意度居全省首位。在全国首创“移动式一窗通办”警务模式，便携式综合办证终端获全国公安基层技术革新大赛一等奖。全民禁毒工程扎实推进。城乡社区网格化服务管理实现全覆盖。安全生产、食品药品安全形势稳定，打私、打假工作扎实有力，有效防范非洲猪瘟等动物疫病，成功应对强台风“山竹”等自然灾害。妇女儿童发展规划实施工作稳步推进。民族宗教领域和谐稳定。军队停止有偿服务所有项目如期完成，征兵和复退军人安置、国防教育、双拥等工作扎实推进。档案、方志等工作取得新成绩。

（六）加强政府自身建设，持续推进治理能力和治理体系现代化。我们落实全面从严治党主体责任，坚持把政治建设摆在首位，大力推进政府系统各方面建设。坚持向市委、市人大及其常委会报告工作，向市政协通报情况，办理建议53件、提案288件，办复率100%。提请市人大常委会审议地方性法规草案2件，制定政府规章2部。深化“大学习、深调研、真落实”工作，形成了12个专题调研报告和系列行动方案。严格执行中央八项规定及其实施细则精神，严厉整治“四风”。加快推进重点领域信息公开和基层政务公开标准化、规范化试点，在全省2017年度政务公开工作评估中居粤东西北地区第二。推进审计管理体制改革，审计监督得到强化。加强干部培训教育和监督管理，打造忠诚干净担当公务员队伍取得新进步。

2018年，我们先后成功举办了一系列重大活动，产生了积极的社会影响和良好的综合效应。举办首届中国农民丰收节分会场活动，成为最精彩、最有影响力的分会场活动。举办柚果产业发展专家问策会，聘请18位院士专家为我市柚果产业发展顾问。举办中国国际绿色创新发展大会，吸引300多家国内外知名企业走进梅州、走进广梅园、对接贫困村。举办南粤古驿道定向大赛平远站活动，挖掘《寻乌调查》内涵，打造红色文化旅游古驿道精品线路。举办首届梅州马拉松赛，1.3万多名国内外选手竞相“亲梅逐马”，被誉为最美最干净的马拉松。举办精准医学研究与应用院士学术大会，借智借力院士资源，高起点建设梅州市医学科学院。举办卡拉比—丘理论发展40年国际会议，“数学国王”丘成桐和海内外顶尖数学家齐聚梅州，搭建了梅州与清华大学合作新通道。举办庆祝曾宪梓博士荣获改革开放40周年“改革先锋”称号大会，弘扬曾宪梓博士倾力支持国家改革开放的精神。举办“迎新年•颂党恩•展新貌”大合唱，广大干部唱出了精气神、唱出了新风貌。

各位代表！过去一年，国际形势复杂严峻，国内经济发展不稳定不确定性因素增多，经济下行压力空前加大。在这样的形势下，能取得这些进展和成绩，确实来之不易。这是我们深入贯彻落实习近平新时代中国特色社会主义思想的结果，是省委、省政府关心重视的结果，是社会各界广泛支持的结果，更是全市上下攻坚克难、团结奋斗的结果。在此，我代表市人民政府，向全市人民、中央和省属驻梅单位、驻梅部队，向所有关心支持梅州发展的港澳同胞、台湾同胞、海外侨胞和社会各界朋友，表示衷心的感谢并致以崇高的敬意！

二、当前的形势和任务

各位代表！回顾过去一年的工作，我们也清醒地看到，我市经济社会发展仍然面临不少困难和问题。总的来看，经济发展不充分仍是我们面临的最突出短板，实体经济发展不足，产业基础薄弱，经济总量小，人均水平低。受国内外经济形势及自身内在原因影响，2018年固定资产投资下降1.5%，一般公共预算收入下降10.6%，企业经营综合成本上升，稳增长任务十分艰巨。一是产业结构比较传统、单一、脆弱，烟草、电力、房地产等产业占比较大，易受调控政策影响，与此同时新产业新动能发展聚集缓慢，应对经济下行压力缺乏多元支撑。二是缺乏大平台、大项目，固定资产投资增长乏力，交通区位条件仍有待改善，自主创新能力有待加强，项目储备特别是产业项目储备不足，影响发展后劲。三是财政收支矛盾突出，财政减收因素较多，而刚性支出不断增加，政府债务及财政挂账历史累积规模较大，化解任务较重。四是资源环境压力加重，环保基础设施欠账较多，查处破坏生态环境的力度有待加大，处理好保护与发展的关系，仍有一系列复杂问题需要解决。五是民生社会事业投入相对不足，基本公共服务均等化水平较低，民生保障能力与群众期望仍有不小差距。六是营商环境处于全省中游水平，公平竞争的机制氛围仍有待加强，对标最高最好最优仍有很大的改善空间。同时，政府作风还需持续改进，政务服务效率有待提高。清醒地认识问题，是我们端正姿态、轻装上阵、阔步前行的基点。剖析这些问题，并不是妄自菲薄、丢掉信心，相反，在过去困难的一年中，我们仍然保持了稳中向好的基本面，我们积累了厚积薄发的众多因素，我们迎来了政策加强的大好机遇。面向未来，我们充满了信心。

从全国来看，我国发展仍处于并将长期处于重要战略机遇期，经济长期向好的发展态势不会改变，作为世界第二大经济体，发展有韧性有底气有后劲。从全省来看，我省经济持续健康发展，连续30年经济总量位居全国第一。省委、省政府高度重视区域协调发展，“一核一带一区”发展格局的构建，为我们带来了新的战略机遇。从全市来看，我们坚决全面彻底肃清李嘉、万庆良恶劣影响，完成中央和省委巡视整改任务，营造风清气正的政治生态；深入开展扫黑除恶专项斗争，打掉一批涉黑涉恶团伙，为项目建设扫除了障碍；持续开展“三好一正”活动，进一步鼓舞士气，形成了干事创业的浓厚氛围。我们坚持聚焦发展不充分问题，加大招商引资力度，主动与一系列国企、民企、外企洽谈对接，引进鼎盛翼天公司打造康养文旅综合体龙头项目，用42天促成广东一一五科技股份有限公司总部搬迁回梅，创造了“梅州速度”，这些必将形成良好的带动效应。我们毫不动摇支持民营经济发展，把广大民营企业家当自己人，大力优化营商环境，在亲清新型政商关系下提供细致、周到的服务，增强了广大企业家的发展信心。

各位代表！2018年，习近平总书记先后四次对广东工作作出重要讲话和重要指示批示，为我省新时代改革开放再出发提供了根本遵循、注入了强大动力。省委十二届六次全会对“1+1+9”工作部署作出了深化安排，省十三届人大二次会议审议通过的省政府工作报告对2019年全省经济社会发展作出了全面布置。市委七届六次全会提出了“123456”思路举措，明确了“六争六补”要求，为我市改革发展、“换道超车”量身订做了施工图。只要我们沉下心来搞建设，扬长补短、科学应对、埋头苦干，就一定能变压力为动力、化挑战为机遇，就一定能战胜各种困难问题，推动梅州振兴发展，开创新局面，取得新成绩。

推动梅州今后一个时期的改革发展，必须立足北部生态发展区定位，按照市委七届六次全会的决策部署，正确处理五个关系，走实走好绿色发展之路，努力实现“换道超车”。必须处理好争优和补短的关系，既要持之以恒把“六争”的优势做得更强，又要大力把“六补”的短板补齐。必须处理好存量和增量的关系，既要抓传统优势产业和本土企业转型升级，优化存量，又要抓招商引资和新兴产业培育，吸引更多的高端增量进入。必须处理好硬件和软件的关系，既要加快改善交通区位条件和配套设施，又要下大力气营造一流的营商环境，增创体制机制新优势。必须处理好内因和外因的关系，既要自力更生、久久为功，强化主人翁意识和履行好主体责任，又要借力省的支持和广州市全方位对口帮扶。必须处理好保护和发展的关系，既要坚持把保护摆在优先位置，夯实生态基础，守好生态底色，决不走“先污染、后治理”的老路，又要抓牢发展第一要务，力戒把生态环境保护当成发展的紧箍咒，把生态发展当成不担当、不作为的托词。通过与“一核一带”的差异化发展，发挥“一区”的后发优势，探索绿水青山转化为金山银山的有效路径，通过产业生态化和生态产业化，在高水平保护中实现高质量发展。

今年政府工作的总体要求是：以习近平新时代中国特色社会主义思想为指导，全面贯彻党的十九大和十九届二中、三中全会及中央经济工作会议精神，全面贯彻落实习近平总书记对广东重要讲话和重要指示批示精神，按照省委十二届六次全会和市委七届六次全会的部署，统筹推进“五位一体”总体布局，协调推进“四个全面”战略布局，坚持稳中求进工作总基调，坚持新发展理念，坚持推动高质量发展，坚持以供给侧结构性改革为主线，坚持深化市场化改革、扩大高水平对外开放，实施省“一核一带一区”区域协调发展战略，落实省委“1+1+9”工作部署和市委“123456”思路举措，主动融入粤港澳大湾区建设，统筹做好稳增长、促改革、调结构、惠民生、防风险、保稳定各项工作，进一步稳就业、稳金融、稳外贸、稳外资、稳投资、稳预期，争先“六项工作”、补齐“六个短板”，打造宜居宜业宜游“世界客都•长寿梅州”，保持经济持续健康发展和社会大局稳定，为全面建成小康社会收官打下决定性基础，以优异成绩庆祝中华人民共和国成立70周年。

今年我市经济社会发展的主要预期目标是：地区生产总值增长5.5%-6%；一般公共预算收入增长4%；固定资产投资增长8%左右，社会消费品零售总额增长8.5%，进出口总额增长3%；城镇新增就业2.5万人，城镇登记失业率控制在3.5%以内；居民消费价格涨幅控制在3%以内；完成省下达能耗双控目标。

三、2019年工作安排

各位代表！2019年是中华人民共和国成立70周年，是决胜全面建成小康社会的关键之年。做好今年的工作，意义重大。

为实现今年的目标任务，我们将重点抓好十一个方面的工作：

（一）突出稳增长，保持经济发展稳中向好

重点抓好投资、消费、进出口，“三驾马车”协同发力，有效应对经济下行压力，促进经济运行保持在合理区间。

全力稳投资。坚持以优质项目比能力、以有效投资论英雄，扎实推进241项年度投资458亿元的重点项目建设。实施五年交通大改善战略，下大力气补齐交通短板，确保梅汕高铁年内建成通车，争取年内动工建设龙梅高铁；保障大潮、丰华、华陆、东环高速无障碍施工，确保梅平高速上半年通车；推动大丰、梅潮、平武高速和梅漳厦高铁、瑞梅铁路、大埔至潮州港疏港铁路、蕉岭铁路货运专线规划建设；梅畲快线和罗乐大桥连接线建成通车；改造提升100公里国省道，启动G205线、G206线梅州城区改线工程；加快梅县机场迁建前期工作。推进水利、能源、环保、信息基础设施项目建设。完成韩江高陂水利枢纽工程年度投资计划8亿元以上。加快建设梅州（五华）抽水蓄能电站厂房和上下库大坝。建设500千伏闽粤联网、500千伏五华输变电工程，全面推进农村电网改造升级。加快建设西气东输三线工程梅州段和粤东天然气主干管网梅州段。推进一批充电站、充电桩规划建设。加快梅兴华丰产业集聚带核心区环保、学校、医院、商业等配套设施建设。突出抓好工业投资，推进105个产业项目建设。推进市中医医院门诊综合大楼、元城小学等56个民生项目建设。

支持扩大消费。把握消费升级趋势，积极培育养老、家政、教育、托幼、文化、体育、信息等服务消费，推动生活服务业多元化发展。挖掘传统消费潜力，支持开展家电、家居建材、食品餐饮等促销活动。落实新能源汽车车辆购置税优惠政策，促进二手车便利交易。努力稳地价、稳房价、稳预期，支持合理自住需求。落实带薪休假制度，促进旅游消费。实施“互联网+流通”行动计划，解决快递服务“最后一公里”问题，加快电子商务快递物流协同发展。修订促进商贸物流业发展规划，建设市级快递物流园区，采取措施降低物流成本。建立健全重要产品追溯体系，加强社会信用和市场监管体系建设，营造安全放心的消费环境。

扩大进出口。落实省“外资十条”“稳外贸九条”，支持丰顺电声、大埔陶瓷等创建国家级外贸转型升级基地，扩大机电、陶瓷、家具等产品出口。主动承接高质量、有市场的加工贸易转移，支持辉骏科技与华硕电脑合作，扩大电脑主板加工出口。组织企业参加国内外展会，抢抓出口订单，开拓新兴市场。鼓励先进技术和设备、重要资源性产品进口。促进市跨境电商清关中心规模化运营，支持跨境说粤东总部基地建设。加快智慧口岸建设，降低通关成本。承接好省下放18项涉外行政许可事项，优化对外企服务，积极扩大利用外资，特别是制造业利用外资。

（二）突出供给侧结构性改革，促进产业高质量发展

坚持“巩固、增强、提升、畅通”八字方针，实施五年实体经济大振兴战略，加快构建“5311”绿色产业体系。

改造提升传统优势产业。争取稳定烟草生产计划，鼓励企业加大高端产品研发，增强品牌优势，更好带动地方经济发展。推进煤电企业清洁生产，争取发电指标，压减生产成本，提高现有产能利用率；发展水电、太阳能项目，优化能源供应结构。以塔牌集团为龙头，扩大高标准水泥产量，发展绿色新型建材和装配式建筑，提升产业链水平。支持电子信息产业向产业链、价值链高端攀升，重点抓好东升工业园转型增效，整合铜箔、印制电路板、电脑主板等产业资源，促进上下游协作配套。支持机电制造骨干企业技术创新，壮大产业规模。狠抓节能减排，严格实行能源和水资源消耗、建设用地等总量和强度双控行动。

加快培育新兴特色产业。做强文旅产业。统筹推进梅江韩江绿色健康文化旅游产业带建设，聚焦山水旅游、生态旅游、红色旅游、新农村旅游等，发展“互联网+旅游”，全力创建国家全域旅游示范市。建设一批国家级旅游度假区等重点项目，中国•梅州“客都人家”康养文旅综合体年内建成实景演出古镇等首期项目，完善旅游交通、游客服务等配套设施，打造国内一流旅游目的地。活化利用南粤古驿道、古村镇、古码头等特色文旅资源，建设一批国家级红色旅游经典景区和文旅小镇，实施乡村旅游“百村示范”工程，促进文旅、农旅融合发展。面向珠三角地区，继续开展“百万人游梅州”活动。发展体育产业。以足球为重点，带动体育赛事、体育运营公司、体育用品制造企业等集聚发展。带着感情认识足球、带着热情服务足球、带着激情振兴足球，制定实施足球振兴新十年规划，开好足球工作大会。办好“两甲一超”主场赛事，兴建足球特色小镇，让小足球踢出大产业，让小足球带出大事业。支持各地“一县一特色”，打造马拉松、自行车、定向越野、山地户外、汽车拉力等体育品牌赛事，形成良好的体育经济生态，拉动体育消费。发展互联网产业和数字经济。加快建设梅州互联网创新中心，出台促进互联网产业发展政策措施，发展大数据、云计算、物联网，支持一一五科技、紫晶存储、帮啦跑腿、村之翼等企业做大做强，建设广梅园大数据产业园、飞翔云计算产业园等平台，努力构建互联网产业发展生态圈。举办梅州互联网产业峰会。推动传统企业应用人工智能、“上云上平台”等。推进信息基础设施建设三年行动计划和十大工程建设，加快4K电视普及和5G商用步伐。

发展大健康产业。依托生态、长寿、富硒等优势，加快发展健康幸福产业。开发森林康养、温泉保健、药膳美食等养生内容，建设紫合医院、东山健康小镇、留隍潮客小镇等一批高品质医养结合项目，引进一批养老养生企业。发展富硒产业，加快广东省质量监督富硒产品和深加工农产品检验站建设，培育发展食品饮料产业，引进一批龙头企业，延伸农产品精深加工链条。加快创建国家中医药综合改革试验区，扩大道地中药材种植面积，发展中药材交易市场、中药饮片和颗粒制造企业，培育中医药全产业链。推动吴清平院士大健康高科技项目、王老吉大健康研究中心、大南药国家重点实验室梅州中心等建设，与葛洪中医药研究院共建中医药产学研合作基地。加强医疗保障，提高人均寿命，积极向国际自然医学会申请认证“世界长寿之都”。

壮大特色现代农业。以现代农业产业园为龙头，深入推进农业供给侧结构性改革，当好广东农业标兵。推动现有省级现代农业产业园提质增效，力争新增3个以上，加快创建国家级现代农业产业园。发展现代种业和设施农业、休闲农业等，扩大农业科技应用，建设农业综合体、农业公园，加快农村一二三产业融合。大力发展“一村一品、一镇一业”特色经济，继续打造梅州柚、嘉应茶、客都米和平远橙等区域品牌，深化与顺丰快递、中粮我买网、中石化易捷便利店、SPAR中国等渠道合作，让好产品卖得更好。制定实施梅州柚产业发展规划，重点调优品种，做强品质。办好中国梅州（丰顺）茶业精英峰会，擦亮茶乡品牌。推广“稻鱼共生”生态种养技术，发展优质稻米。继续举办中国农民丰收节相关活动，办好中国柑桔学会2019年学术年会。

（三）突出抓项目抓企业抓平台，进一步夯实发展条件

树立“产业兴市、项目为王、企业第一”理念，狠抓项目建设，大力培育市场主体，推进平台载体建设。

抓好产业项目建设。建立完善产业项目建设工作和考核机制，吸引更多优质项目落户，形成“储备一批、动工一批、建成投产一批”的良性机制。加强项目用地用林、市政配套、环评审批、资金落实等方面统筹，落实新上重大项目能耗指标实行单列，确保重大项目按时落地实施。因地制宜发展绿色低碳新型工业，加快绿十字生物科技等一批项目动工，促进广汽零部件、广药王老吉等一批在建项目投产达产，支持BPW车轴、博敏电子等一批增资扩产项目建设，支持有条件的企业加快技术改造。

加强企业培育。依托广东一一五科技股份有限公司、广东三角牌电器股份有限公司等，培育产值超百亿元企业和上市公司。新增一批10亿元、5亿元以上骨干企业，加强企业后备梯队建设。通过建设电声小镇、发展印制电路板产业联盟等，培育若干个产值超百亿元的产业集群。支持上市企业扩大本地投资，做大产业规模，推动更多优质企业到科创板、新三板等上市，提升企业直接融资额度。扶大扶强本地建筑企业，引进优质建筑企业。支持五华县申报“中国建筑之乡”。加强科技企业孵化器和众创空间建设，孵化一批“双创”企业，支持成长性良好的中小微企业解决发展瓶颈问题。积极开展“保规上”“育上规”，推动一批“个转企”，力争全年“小升规”65家以上。

发展壮大民营经济。放宽和规范市场准入，按照“法无禁止即可入”扩大民间投资，引导民营企业通过规范的PPP模式等途径，参与基础设施和公共服务领域建设。认真贯彻国家和省对增值税小规模纳税人“六税两费”减按50%征收等减税降费政策，全面落实省“实体经济十条”、市“实体经济十二条”，专项治理政府部门下属单位、行业协会商会、中介机构等收费行为，多渠道减轻企业负担。推行“首席服务官”制度，建立政府与企业“双月”定期沟通机制，帮助企业解决融资、用地、用工等方面问题。加大力度查处损害企业权益行为，营造公平竞争环境，激发和保护企业家精神。搭建政银企对接平台，支持各商业银行和法人银行开展服务中小微企业信贷业务。做实信贷风险补偿资金等三大融资平台，逐步降低融资成本。

做强发展平台。实施五年平台大建设战略，加快补齐发展平台短板。开展高新区提升行动，争取早日升级为国家级高新区。按照产城融合、区域整合的原则，大胆革新广梅园管理体制，打造广州梅州改革举措、政策双重叠加的“特区”。加快推进广梅园绿色创新中心、畲江现代产业孵化园、广铁物流园等建设，力争梅州综合保税区年内获批。理顺东升园管理体制，推进西阳片区、城北片区规划建设。促进蕉华园与蕉岭县融合发展、产城互动。加快各县（市、区）工业园区特色化发展，创建一批省级高新技术开发区。按照“点上开发、面上保护”原则，推动企业入园，加强开发强度管控，把好环保准入门槛，中心城区、县城及各类省级以上重大发展平台和开发区，适用省重点开发区域和东西两翼地区的发展政策。加强土地储备，提高批而未供和闲置土地处置率。

狠抓招商引资。制定大招商三年行动方案，完善招商激励机制，压实各级各部门招商责任。组建招商团队，重点在广深和京沪杭等地举办系列专题招商活动，突出精准招商、产业链招商，致力引进一批医药、绿色食品、新材料等制造业项目和大文旅、大农业、大健康等项目。实施“乡贤回乡投资兴业工程”，吸引更多梅州籍客商回乡投资。引进平台型企业，建设工业产业承接平台，实施以商招商。

（四）集中力量攻坚克难，推动三大攻坚战取得决定性进展

今年是三大攻坚战承上启下的重要一年，必须针对突出问题，打好重点战役，为2020年胜利收官打下决定性基础。

防范化解重大风险。针对当前突出的财政收支矛盾，树立长期过紧日子的思想，除刚性和重点项目支出外，一般性支出一律按不低于5%的幅度压减，“三公”经费只减不增。按照“严控增量、化解存量”要求，规范政府举债融资行为，坚决遏制隐性债务增量。规范财政资金管理，各级财政暂付挂账一律压减15%以上，对各预算单位结余资金以及结转两年以上的资金一律收回。全力防范化解金融风险，严厉打击网络电信诈骗、非法集资、涉黑涉恶洗钱等违法行为。坚持“房子是用来住的，不是用来炒的”定位，深化租购并举的住房制度改革，科学调控房地产市场，防止房价大起大落。

深入推进精准脱贫。整合创新扶持政策，深化到村到户到人的精准分类施策，更加关注和支持贫困村外分散的贫困人口，确保全面小康路上不漏一户、不落一人。充分发挥各级驻村扶贫工作队作用，加大产业扶贫力度，让每个有劳动力、有参与意愿的相对贫困户都有扶贫产业带动。组织贫困人口参加客家菜师傅、建筑工匠等职业技能培训。做好教育、医疗、住房等保障性扶贫，使低保政策与扶贫开发政策有效衔接。健全脱贫长效机制，解决因病返贫、因学返贫、因灾返贫等问题，确保长期稳定实现“两不愁、三保障、一相当”目标。

推进铁腕治污。坚决打好蓝天、碧水、净土三大保卫战，确保全年空气质量优良天数达到98%以上、饮用水源水质达标率100%、国考和省考断面水质优良率100%。落实主动减量要求，对“三高”产业果断淘汰。建立“散乱污”工业企业（场所）清单并实行分类综合整治，建设机动车遥感监测系统，推进挥发性有机废气“一企一策”治理，建立市县镇村露天焚烧网格化监管机制，落实扬尘管理“六个100%”要求。全面落实河（湖）长制，深入开展“五清”专项行动，打造具有梅州特色的“碧道”建设模式。实施梅江入河排污口“身份证”管理。建成黄塘、周溪污水处理厂。开展县级及以下集中式饮用水水源地保护区环境问题清理整治，对城区自来水水质加强监测。适度超前规划建设环保基础设施，完成中心城区、县城污水处理厂配套管网和镇级污水处理设施建设，规划建设县级生活垃圾无害化处理设施，完成镇级填埋场整治。编制危险废物污染防治规划，推进垃圾焚烧发电、固废和危废、建筑垃圾处理设施扩能或新建。加快推进兴宁市静脉产业园建设。争取开展“无废城市”建设试点工作。逐步推进城乡生活垃圾强制分类，建立分类投放收运处理体系。确保农村生活垃圾收运处理率达100%。严格控制农业面源污染，落实限养禁养区划，引导生猪养殖上大压小、规模化发展，年内完成各县（市、区）畜禽无害化处理中心建设。

（五）实施创新驱动发展战略，提高科技创新能力

充分发挥创新第一动力作用，开展五年科技创新上水平行动，完善区域自主创新体制机制，支撑我市经济高质量发展。

建设创新平台。重点建设“三院一基地”。推动市医学科学院开展实质性运作，加强与院士团队合作，争取在重大疾病诊断治疗、预防控制等方面取得研究新突破。组建市农业科学研究院，有效整合各类涉农科研机构，形成研究合力，促进农业科技创新和成果转化。共建梅州产业技术研究院，深化与省科学院合作。依托仲恺农业工程学院，加快建设仲恺（广梅园）教学实践和科技成果转化基地。

狠抓重点领域创新和技术转移。围绕我市重点产业和社会发展需求，支持龙头企业、科研院所等开展技术攻关，力争在一批共性技术、关键技术上实现突破。探索建立印制电路板、电声等行业创新联盟，提升产业链协同创新水平。加强农作物优良品种选育，加快柚果等农产品深加工技术研发及引进，争取更多微生物育繁推技术落地应用。建设技术转移中心和知识产权运营平台，扩大先进适用技术交流推广。大力发展工业设计产业，促进制造与设计融合。深化科技体制改革，集中科技资金投向。创新形式，推动科技与金融相结合。开展“双创”活动，培育一批省、市级小型微型企业创业创新示范基地。

发挥企业创新主体作用。深入实施高新技术企业树标提质行动，培育一批具有自主知识产权和核心技术的科技型企业，高新技术企业达200家以上。推进产学研深度融合，鼓励支持企业培育和引进省级以上工程中心、技术中心、院士工作站、孵化器、公共科技平台等，力争规上工业企业设立研发机构比例提高到35%以上。开展形式多样的协同创新，支持重点企业与国内外高校、科研机构开展产学研合作，组建新型研发机构。

打造创新人才高地。落实“人才新政20条”和金融人才政策，推进“柔性引才”，抓好世界客属青年创新创业中心、院士团队创新创业（梅州）驿站等平台，吸引创新团队进驻。实施急需紧缺人才引进计划、千名企业家培养工程。实施文化名家暨“四个一批”骨干文化人才培养工程、文创人才培育工程。完善人才服务保障体系，着力解决人才落户、住房、子女就学等问题，让人才引得进、留得住。

（六）推进区域协调发展，构建“五星争辉”格局

坚持高起点谋划、高质量建设、高标准管理，打造“三宜”城市范例，推动中小城镇发展，加快以人为本的新型城镇化。

优化区域功能定位。构建以广梅园为地理联结点的半小时经济圈和高密度城镇群，支持梅江、梅县、兴宁、五华和高新区打破行政区划，加快交通基础设施、生态环境保护、产业、规划布局等一体化建设，瞄准未来大城目标，着力打造新型工业化、新型城镇化深度融合的经济增长“大星”。支持平远、蕉岭、大埔、丰顺打造县域特色发展“四星”，完善基础设施和公共配套，发展绿色低碳新型工业和文化生态旅游、健康养生、长寿食品、现代农林业等产业，做大做强县域经济。

推进中心城区提质发展。围绕建设“三宜”城市，开展中心城区总体城市设计研究，加快推进控制性详细规划编制全覆盖。推进嘉应新区起步区完善功能，基本建成主要道路管廊、绿轴广场和“两馆一场”等配套设施，谋划江南新城南拓方案。巩固“美丽梅州•美好家园”城乡环境大提升行动成效，拆除违法建筑，打通市区人民南路、新峰路、泮坑公园通道等一批断头路，兴建一批人行天桥、小公园、停车场、集贸市场、社区体育公园等市政项目，加大力度治理交通拥堵、内涝等“城市病”。扩大梅花、桂花等苗木种植，努力建设花园城市。抓好历史文化街区保护性开发，挖掘名人故居资源，以“绣花”功夫推进老城区“微改造”，改造建设金山顶小公园、百岁山休闲健身公园等项目，打造老城新貌。开展群众性“最靓屋夸”创建活动，建设与邻为伴、与邻为善、守望相助的社区生活共同体。推进国商中心盘活利用，加快望江楼、凯旋门等历史遗留问题解决步伐。加强城市管理，推动智慧交通、智慧安保等建设，促进大数据在市政领域应用，提升智慧城市水平。

加快县城和特色小镇建设。推动南部人口大县加快县城新区建设，有序扩容提质，吸引农村人口进城落户，提高户籍人口城镇化率。统筹推进重点镇行政管理体制改革，探索在雁洋、华城、安流、留隍、茶阳等较大镇创新体制机制，赋予相关县级管理审批权限，打造联城带乡的协调发展节点。推进潮客小镇、足球小镇、花灯小镇、珠宝小镇、物联网小镇、温泉小镇、南药小镇等创建对象规划建设，新规划创建科教小镇等一批市级以上特色小镇，突出主导产业，完善综合功能，开展生产生活生态融合发展示范，引领城乡区域创新协调发展。

（七）强力实施乡村振兴战略，提高城乡发展协调性

坚持农业农村优先发展，发挥梅州农业农村的特色优势，推动“五大振兴”，下力气解决城乡二元结构问题，促进农业强、农村美、农民富。

促进农民增收。实施“提升百家新型农业经营主体”工程，争取全年培育省级重点农业龙头企业10家以上，培育一批种养大户、新型职业农民，完善农业专业人才引进政策。创新农村土地流转方式，引导工商资本下乡。发展林下经济，加快国家储备林项目建设，培育2家国家级、6家省级林业龙头企业。争取更多产品获得国家农产品地理标志、国家森林生态标志产品认证。推进冷链物流、商贸流通标准化建设。推进农村建设用地拆旧复垦，完成水田垦造任务，把农村闲置资源变成可交易资产。实施“客家菜师傅”工程，发展壮大农家乐、民宿客栈，推动客家菜产业化，培训“客家菜师傅”1000名。推进高标准农田和粮食仓储建设，保障粮食安全。继续防范非洲猪瘟等疫病。

改善农村发展条件。深入开展“千企帮千村”活动，全力补齐乡村道路、水利、信息、农网改造等基础设施和公共服务短板。加快“四好农村路”建设，全面整治通建制村“畅返不畅”路段，改造农村公路四、五类桥梁。实现20户以上自然村光纤全覆盖。完成158.5公里山区中小河流治理任务。实施农村饮用水安全巩固提升工程。完成村庄规划编制，强化宅基地和农村建房规范管理。深入推进厕所革命，农村无害化户厕普及率达到98.5%。推进全域农村人居环境整治，基本完成村庄“三清三拆三整治”，深入推进省定贫困村创建新农村示范村，巩固省级新农村连片示范工程，多村联动开展“农村景区化”改造，申报建设省际廊道美丽乡村建设，打造岭南最美乡村。

深化农村综合改革。实施乡村振兴综合改革试点，扎实推进培育新型农业经营主体、完善农村土地制度和集体产权制度、农村“三变”、涉农资金整合、农村项目审批管理等重点领域改革，着力解决制约“三农”发展的深层次问题。深化供销社改革创新，提升为农服务能力。总结推广农村“两权”抵押贷款试点模式，促进普惠金融发展。

（八）深化重点领域改革，打造优质投资发展环境

以庆祝改革开放40周年为动力，进一步解放思想，全面深化改革攻坚，充分释放市场潜力和社会活力。

加快行政体制改革。高质量完成市、县机构改革，优化职能配置，提高政府部门行政效能。全面推开市、县承担行政职能的事业单位、生产经营类事业单位分类改革，实现政事分开。深化强县放权等“放管服”改革，继续向县下放一批市级管理权限，再压减市级职权事项，优化权责清单，更好厘清市县间权责关系。开展“百项疏堵行动”，持续深化减证便民。

加快经济体制改革。推进国有资产监督管理体制改革，全面完成市级国有资产的清产核资，推进“小散弱”国有企业整合，组建基础设施、工业、文旅等领域国有企业集团；整合所有经营性资产、平台公司，成立国有资产运营公司。基本实现国有“僵尸企业”市场化出清。加强非经营性资产管理。深化要素市场化配置改革，推进要素市场建设，保障市场体系竞争有序。

加快营商环境综合改革。实施企业投资项目分类管理和落地便利化改革，提高主动服务企业的意识和能力，围绕工程建设项目审批、企业开办注销、不动产登记、用水用电用气等开展专项行动，率先在粤东西北地区实施企业开办全免费，实现开工前政府审批时间民用建筑项目不超过41个工作日、工业企业不超过45个工作日，力争营商环境进入全省前列。加快构建线上线下一体融合的公共政务服务体系，深化“数字政府”改革建设，加快各级各部门政务系统迁移上云，推动政务数据开放共享，推进各级实体办事大厅、服务站点标准化建设，年底前重点领域实现“一门、一网、一次”办理。推动更多高频服务事项入驻“粤省事•梅州”指尖办理，已上线服务事项“零跑动”超过80%。

推进生态文明改革。加快建设享誉全国的生态高地。建立“三线一单”环境管控体系，强化“三线”管控为保障的国土空间开发保护体系，确保重要生态空间的生态功能不降低、面积不减少、性质不改变。对照国家公园创建标准，新建或提升改造一批国家森林公园。加强森林抚育，实施崩岗等水土流失治理、“牛肝土”山水林田湖草综合治理，加强损毁山体、废弃矿山地质环境和生态修复，筑牢粤北生态屏障。推动森林进城围城，优化街头绿地公园，确保成功创建国家森林城市。强化生态文明建设目标评价考核结果公开运用，大力发展绿色金融，探索建立绿色GDP核算体系和生态激励型财政机制。构建跨界流域联防联控联治机制，争取建立韩江流域上下游横向生态补偿机制。

建设中国足球特区。围绕打造国内一流足球城市，积极申报建立国家级平台“中国足球特区”。推进足球体制改革，重点抓好足协实体化、青训体系、校园足球、外聘教练、运动员入学等改革，完善足球运动中心运作、场馆运营、场地建设等机制。推进国家青少年训练基地建设。筹建高水平足球学院，培育足球专业人才。

（九）扩大对外开放，加快构建对外开放新格局

坚持以开放促发展，打开山门、敞开围龙，用好国内外两个市场、两种资源，在更高水平上扩大开放。

主动融入粤港澳大湾区建设。抢抓机遇，对接粤港澳大湾区建设发展需要，出台行动方案，在农产品供给、休闲旅游、公共服务、青年创业等方面，主动参与大湾区产业分工合作，积极承接大湾区产业技术转移。复制推广自贸试验区改革试点经验，探索与前海等自贸片区构建协同发展机制。密切与深港创新圈合作，加强人才培养和科技引进。

扩大对外交流合作。加强与“一带一路”沿线国家和地区的经贸、文化、体育、旅游等往来，继续办好梅州五华国际女子足球锦标赛、“一带一路”客家杯足球邀请赛。加强海外经贸文化联络机构建设，在海外举办投资推介会。用好侨资侨力，推动与我市对外开放发展互补性强的国家地区建立友好城市。重点开拓与新加坡政府的合作，争取合作事项列入新加坡——广东合作理事会议事日程。依托松口、雁洋、丙村在侨界的影响力，推动梅州华侨文化旅游经济合作试验区规划建设。办好第六届世界客商大会。

提高开放融通水平。积极推进与粤港澳大湾区交通基础设施互联互通，尽早融入广州深圳2小时经济圈，有序加密或开通北京、上海、深圳及香港、新加坡等航班航线。依托汕昆高速、即将通车的梅汕高铁，以及待建的梅潮高速、大埔至潮州港疏港铁路等，规划建设梅汕、梅潮经济走廊，支持东部的丰顺、大埔对接融入沿海经济带建设，推动梅州借道出海、向东发展。办好赣闽粤十三市党政领导联席会议，务实推进生态环境和旅游合作，构建梅（州）赣（州）、梅（州）龙（岩）跨境水治理协作机制，共同推动规划建设梅漳厦高铁，联通厦漳泉城市群，更好地融入海西经济区建设。

（十）加快文化名城建设，推动物质文明和精神文明协调发展

高度重视发展文化软实力，在文化名城建设中焕发客家文化的生机活力，不断提升人民文明素养和社会文明程度。

推进全国文明城市创建。实施梅州精神文明建设提质三年行动计划，突出创建常态化，深化城乡“四级联创”。以新时代文明实践中心建设为主抓手，有序整合县级资源，全面提升志愿服务质量，创新开展新时代群众工作。大力培育和践行社会主义核心价值观，广泛开展弘扬时代新风行动，深入开展文明出行、文明交通、文明旅游、文明餐桌等活动，深化群众性文明创建。

繁荣发展文化事业和文化产业。加大公共文化服务投入，完善市县镇村四级公共文化服务体系建设。加强文化遗产保护工作力度，建立客家文化（梅州）生态保护实验区数据库，建设一批非遗展示馆、非遗代表性项目传习中心和传习所等，办好客家文化（非遗）艺术周。加强对重点文物、革命遗址、客家古民居、古村落及名人名居的保护开发，引入社会力量多种形式活化利用。升级改造中国客家博物馆，推进中央苏区（广东）历史博物馆建设。实施文艺精品创作工程，振兴文艺院团，围绕“庆祝中华人民共和国成立70周年”，力争全年推出2-3部精品剧目。开展文化惠民，提升阅读指数，打造“书香梅州”。规划建设梅州传媒文化创意产业园，加快形成特色文化创意产业集群。加快县级融媒体中心建设。积极发展智慧广电、数字出版、网络文化服务等文化新业态。办好第七届客家文化旅游创意产品博览交易会。进一步加强新时代地方志工作。

（十一）发展社会各项事业，加强保障和改善民生

顺应人民群众新关切新期盼，加快补齐民生短板，不断增强群众的获得感、幸福感和安全感。

办好十件民生实事。主要包括提高底线民生保障水平、扶持创业就业、促进教育更加公平均衡、提升医疗卫生服务能力、实施文化惠民工程、关爱特殊群体、完善城区公共服务设施、改善农村生活生产条件、保障公共安全、加大政务便民服务力度等。

加强社会保障。坚持就业优先，加强高校毕业生、农民工、退役军人等群体就业服务，加大援企稳岗力度，做好困难人员托底帮扶，确保“零就业”家庭动态清零。强化“智慧社保”建设，落实全民参保计划，提高基本养老保险、基本医疗保险覆盖面，推进医保支付制度、社保精准扶贫等改革，完成城乡医保市级统筹。提高城乡低保、特困人员供养和医疗救助标准，加强特困供养、救助、托养等机构管理，把政府兜底的责任履行好。做好农村留守儿童和妇女、老年人的关爱服务，加快养老服务事业健康发展。

推进教育振兴。开好全市教育大会。深化教育综合改革，出台教育振兴五年行动计划，建成“广东省推进教育现代化先进市”。制定城区住宅小区配套教育设施建设与管理工作的实施办法。提升公办幼儿园和普惠性民办幼儿园学位覆盖率，推动义务教育优质均衡发展，全年新增公办幼儿园学位2000个、义务教育学位4170个，解决城区大班额和农村薄弱学校的突出问题。建立学前教育、公办普通高中生均拨款制度及逐步增长机制。适应新高考改革，完善高中教育的设备设施和招生、教研、培优激励机制，促进东山中学等示范性高中提高教学质量。加快教育信息化，推动优质教学资源共享。支持社会力量投资办学，鼓励发展国际化教育。办好特殊教育。整合职业教育资源，创建“广东省职业教育综合改革示范市”，加快南方紫琳职业学院建设，启动梅州市技师学院升格高职院校、兴宁市技工学校升级高级技工学校。支持嘉应学院实施“粤东西北高校振兴计划”，深化与中山大学合作，推进省市共建，建设“国家教师教育创新实验区”。减轻学生负担，规范校外培训，重视校园安全，加强青少年心理健康辅导。积极推进教师“县管校聘”管理改革，加大奖教奖学力度，加强学校班子、教师队伍和师风师德建设，培育名校长、名教师，让名校有影响、名师有地位、教师有尊严。

建设健康梅州。深化医药卫生体制改革，以建设紧密型医联体为抓手支撑分级诊疗制度，推进药品跨区域联合集团采购，实施按病种分值付费，抓好基本公共卫生服务项目。支持市人民医院早日建成广东省高水平医院，加快市中医医院以及兴宁、五华、丰顺等有条件的县（市）创建三甲医院，打造粤闽赣边医疗高地。动工迁建市妇幼保健院，加快县级医院、乡镇中心卫生院升级改造。加强基层卫生人才队伍建设，培养急需的儿科、产科、全科医生。实施中医药传承创新发展行动计划，进一步提升中医药服务能力。完善疫苗管理，做好出生缺陷综合防控，加强妇幼健康服务。深入推进国家卫生县城创建，落实疾病防控措施，实施最严格的食品药品安全监管制度，保障公共卫生安全。

建设平安之乡。以“对标一流、赶超先进”为目标，推进平安梅州建设，努力把梅州建设成为全省最安全稳定、最公平公正、法治环境最好的地区之一，打造平安之乡。深入推进扫黑除恶专项斗争，严厉打击各类违法犯罪。加强公安派出所等政法基层基础建设，强化智慧新警务建设。加快建立网络综合治理体系，推进依法治网。加强国家安全工作。深入推进全民禁毒工程。创新社会治理，打造“枫桥经验”的梅州样板。完善“中心+网格化+信息化”平台建设，推动社会治理重心下移。加强和完善城乡社区治理，规范村务监督委员会建设，实现村民理事会全覆盖，以社会主义核心价值观引领修订完善村规民约，塑造乡村文明新风。落实安全生产责任制，持续开展道路交通、消防等重点领域专项整治，强化应急处置能力建设，提高防灾减灾救灾水平。夯实人民信访基层基础力量。强化法治梅州建设，深入推进法治建设“四级同创”，深化一村（社区）一法律顾问工作，推进“七五”普法工作。依法加强民族宗教事务管理。大力支持国防和军队建设，突出抓好国防动员、国防教育、拥军优属、军民融合等工作，高质量建立健全退役军人服务管理体系和保障机制，维护军人军属合法权益，力争我市创建“全国双拥模范城”实现“三连创”，营造全社会尊重退役军人、尊崇军人职业的良好氛围。

各位代表！做好今年经济社会发展各项工作，必须加强政府自身建设。在新的一年里，我们将坚定不移加强政府系统党的建设。旗帜鲜明讲政治，始终坚持以习近平新时代中国特色社会主义思想为指导，持续开展“大学习、深调研、真落实”工作，树牢“四个意识”，坚定“四个自信”，坚决做到“两个维护”，涵养风清气正的良好政治生态。我们将加快建设法治政府。全面落实行政执法责任制，推进行政复议体制机制改革，严格公正文明执法。加强重点领域立法及宣传，强化规范性文件监督管理，推进依法治统和审计监督全覆盖，推动政府工作全面纳入法治轨道。我们将切实改进作风。把2019年定为“转作风、抓落实”之年。弘扬苏区精神，传承红色基因，发扬逢山开路、遇水架桥的改革精神和闻鸡起舞、日夜兼程、风雨无阻的工作劲头，坚持“马上就办、办就办好”和“官不避事、事不避难”，提高政府执行力。畅通联系群众渠道，整治庸政懒政怠政和不作为、乱作为。严格落实中央八项规定及其实施细则精神，集中整治形式主义、官僚主义。我们将坚持廉洁从政。严格履行党风廉政建设责任制，增强斗争精神，持之以恒正风肃纪反腐，坚决整治群众身边的不正之风和腐败问题。加强廉政文化建设，深入推进政府系统模范机关创建，打造忠诚干净担当的高素质公务员队伍。

在新的一年里，我们将坚持向市委、市人大及其常委会报告工作，支持市政协履行职能，主动加强与各民主党派、工商联、无党派人士和人民团体的沟通协调，广泛接受社会监督，提高代表建议和委员提案的办理质量，确保政府科学决策、民主决策和依法决策。

各位代表！时代是出卷人，人民是阅卷人。面对时代的召唤、人民的期盼，唯有实干二字。不干，半点马克思主义也没有。蓝图已经绘就，让我们更加紧密地团结在以习近平同志为核心的党中央周围，高举习近平新时代中国特色社会主义思想伟大旗帜，认真落实省委“1+1+9”和市委“123456”工作部署，同心同德、迎难而上、真抓实干，以新时代新担当新作为努力开创梅州发展的新局面！
